# 

**DOI:** 10.1017/S0963180122000032

**Published:** 2022-10

**Authors:** Amer Raheemullah

**Affiliations:** Department of Psychiatry and Behavioral Sciences, Stanford University School of Medicine, Stanford, California 94305-5119, USA

Dopamine Nation carries an extremely important message for all of us trying to understand and navigate our “hypermedicated, overstimulated, pleasure-saturated world.” We are all affected to some degree by the compulsive overconsumption of social media, online entertainment, chemically enhanced foods, and pornography. Stanford Psychiatrist Anna Lembke, MD, explores how our digital devices have set off a major societal shift by creating widespread and immediate access to a variety of highly rewarding stimuli. She equates them to the modern equivalent of the hypodermic needle, ready to deliver our choice of digital drug. Similar to chemical drugs, she describes how our compulsive overconsumption leads to regret when we break our own rules, isolation as they replace relationships, and progression of the pain we are trying to escape. These technological advances have outpaced our ability to cope with them and find balance. The book gives us a moment to pause and process how we gradually ended up here, and then takes us through what we can do about it.

Lembke points out the curious fact that we are more depressed and unhealthy than ever, despite all of our medical, psychological, and technological progress. Even more surprising, much of this is a consequence of behavior that can be modified voluntarily, but we choose not to. Lembke articulates the problem convincingly and clearly, using our contemporary language—science. She draws from neuroscience, behavioral economics, psychology, and others to describe timeless universals on balance. She focuses on dopamine, the main neurotransmitter involved in reward and motivation, and tells the story of how habits work. She simplifies complex neuroscience—getting to the bottom line of what you need to know to help with your overconsumption. It spares the reader from the neuroscience jargon and debates, using concise examples and fun pictures to drive home actionable information.

One of the clever aspects of the book, that sets it apart from others in the genre, is the story-telling. It is gripping and sometimes provocative. It knows its audience. It is just the type of story-telling needed for the mind wired for immediate gratification—it provides enough novelty to channel our compulsive reward seeking toward the help in the book. Using her decades of experience as a psychiatrist and addiction specialist, she tells stories of her addicted patients—their pitfalls and successes. She uses patients with addiction as a model for us, since they have already adapted methods to break free from highly rewarding stimuli, and can teach us to do the same. These stories are relatable and inclusive of people from various backgrounds, which is a rare quality, and unique feature of the book. Lembke even uses her own story of compulsive overconsumption to show how these tools that are strong enough to break severe addictions, can also be practically applied to our everyday overconsumption. The stories are authentic, raw, and honest to the emotion behind these struggles.

A major strength of the book is how it addresses society’s role in dealing with the problem of overconsumption. This is too often a forgotten part of the conversation. In addition to strategies for individual improvement, it is crucial to address societal factors, and those most vulnerable to them. The book confronts this tactfully, and with the confidence of its real-world experience. It acknowledges the dramatic increase in widespread access to highly rewarding stimuli as potentially the most important risk factor for unhealthy overconsumption. By way of example, it discusses how the increase in cigarette production led to increasing nicotine use, and the wide-spread distribution of opioids led to the current opioid epidemic. While on the other hand, the public regulation of unhealthy substances like alcohol is discussed, for example through Prohibition’s ability to regulate overconsumption and improve health outcomes. Additionally, the book touches on class inequality, wealth distribution, social upheaval, and their important roles in the conversation. It also cautions against solely medicating the consequences of these problems, which can constitute a type of institutional neglect and abandonment, if psychosocial determinants of health and basic needs are not also met. It suggests alternative, healthier visions of community and society through prosocial shame which demands empathy, acceptance, and radical honesty.

The book goes on to give clear answers, step-by-step, on how to curb or stop unwanted overconsumption and addictive behaviors. It is as if we are inside Lembke’s office receiving the personal attention and guidance of a seasoned provider. The book almost acts as a surrogate for the majority of people who do not have access to this type of specialized behavioral treatment. The techniques are based on the latest science on behavior change, the brain, and psychology, and guided by what has actually worked in the clinical setting. Also included are conventional, time-tested wisdom from the 12-step program, adapted to deal with everyday compulsive habits. All of the tools discussed in the book are practical and easy to start using immediately throughout the day.

I enjoyed the quick pace and easy read of the book, moving from philosophy, science, and patient stories. Lembke has a skill for distilling complex concepts into easy-to-understand explanations. All of this is organized to keep the book fresh and difficult to put down. Yet it all serves the ultimate purpose of the book to frame the problem and provide solutions that are practical. This is an essential read for people struggling with unwanted, excessive behaviors related to anything from social media, smartphones, and food, to those struggling with addictions from pornography, gambling, or drugs and alcohol. It also would benefit anyone wanting to understand more about the brain, habits, and compulsive overconsumption, in order to help navigate this new world for themselves and their loved ones. Finally, I would recommend it to others in the behavioral health field. It models different strategies to implement behavior change, contains a collection of simple explanations for difficult concepts, and the notes at the end provide a roadmap for deeper study. Lembke is an original and powerful voice, providing much-needed clarity and guidance for our overstimulated world.

